# Utilization of pedicled buccal fat pads for coverage of the lateral relaxing wound: A review of literature and a case series of 15 patients

**DOI:** 10.4317/jced.54797

**Published:** 2018-05-01

**Authors:** Muhammad Ruslin, Andi S. Hajrah-Yusuf, Andi Tajrin, Lun-Jou Lo, Tymour Forouzanfar

**Affiliations:** 1 Department of Oral and Maxillofacial Surgery Faculty of Dentistry University of Hasanuddin, Makassar, Indonesia; 2 Department of Oral and Maxillofacial Surgery/Oral Pathology, VU University Medical Center/Academic Center for Dentistry Amsterdam (ACTA), Amsterdam, The Netherlands; 3 Department of Plastic and Reconstrutive Surgery Chung Gung Memorial Hospital, Taoyuan, Taiwan

## Abstract

**Background:**

The buccal fat pad (BFP) is an encapsulated mass originated from a specific fat tissue in various volume throughout the life of each person and BFP has been used in various surgeries as a source of useful graft material due to its easy accessibility and rich vascularization.

**Case Report:**

This report describes fifteen patients who were treated with buccal fat pads (BFP) as a pedicled graft for lateral relaxing wound closure in primary cleft palate surgery. A review of relevant literature is also presented.

**Results:**

All patients had a mean follow-up of 3.7 weeks with a minimum follow-up time of three weeks and a maximum follow-up of four weeks. All patients who had an uneventful immediate postoperative period showed signs of BFP epithelialization characterized by a yellowish tissue beginning in the first week and ending within 3–4 weeks after surgery.

**Conclusions:**

The use of BFP for the small to medium-sized defects reconstruction in palatoplasty is a safe and reliable method with fast healing. Even older patients who would not be able to tolerate time-consuming flap reconstruction procedures had good results.

** Key words:**Cleft palate, buccal fat pads, lateral relaxing wound.

## Introduction

The buccal fat pad (BFP) is an encapsulated mass originated from a specific fat tissue in various volume throughout the life of each person (1). The BFP is located among the masseter and buccinator muscles, ascending ramus of the mandible, and the zygomatic arch (2). Since 1977 BFP has been used in various surgeries as a source of useful graft material due to its easy accessibility and rich vascularization (1,2).

Nowdays, BFP can be used as a packing material to cover the cleft palate relaxing incision for the reconstruction of palatal cleft (3). Although promising results from the use of BFP in palatal cleft surgeries have been published in various literatures (4-8), the procedure is either not a common practice or is under reported in Indonesia. In addition, no literature has been published providing the outcome of techniques already used especially in wider defect and hereby supporting the choices of the Indonesian cleft surgery team.

This study highlights the use of BFP as a pedicled graft for covering the lateral relaxing wounds during primary palatoplasty procedure in various age of patients. A review of the literature from around 100 cases of BFP utilization was also performed ( Table 1).

**Table 1:**
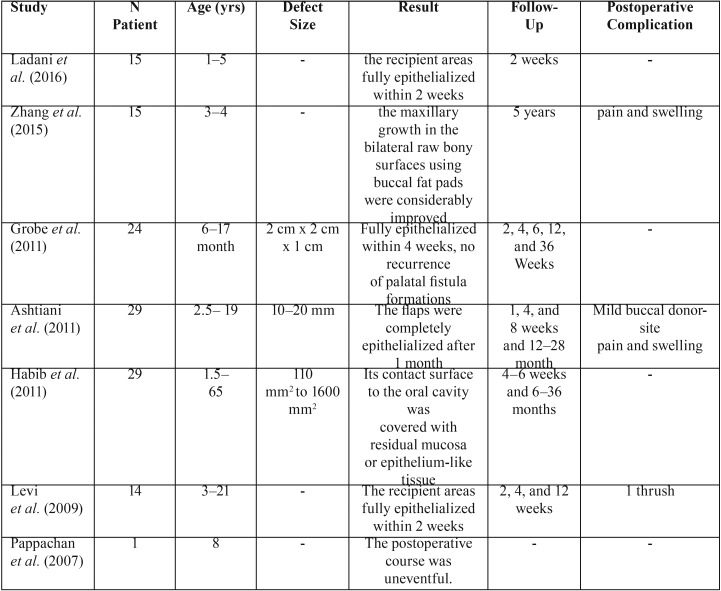
Previous Literature have proved a good result with utilization of BFP for coverage of the lateral relaxing wound.

## Case Report

In the present study, twenty-nine patients underwent palatoplasty procedure selected from January to February 2016 but only 15 patients (seven male and eight female) were treated with BFP as pedicled graft in their operation. The criteria of patients that selected to receive buccal fat pad were wide palatal clefts (≥ 1 cm × 1.2 cm) and vertical palatal shelves whether complete, incomplete or isolated. Patients with narrow clefts, platelet dysfunction, history of previous surgery and previous scar were not selected in the operation. The age of the patients was ranging from 1 year to 32 years old (mean age was eight years old). Eight patients had bilateral cleft palate and seven patients had unilateral cleft palate ( Table 2).

**Table 2:**
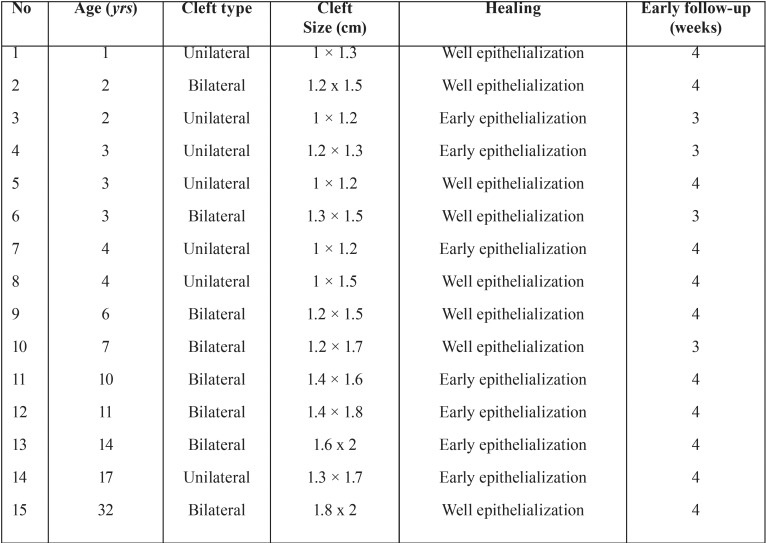
Patient characteristics, cleft palate description, and post-reconstruction outcome of patients in current series.

Ethical approval was granted by the Health Research Ethics Committee of the Faculty of Medicine Hasanuddin University, Makassar, Indonesia. The palatal cleft was reconstructed using the double opposite z plasty Furlow technique, and was performed by single experienced oral and maxillofacial surgeon (MR) under general anesthesia with oral intubation.

Before surgery, all patients were given antibiotic prophylaxis and maintained on soft food diet. They underwent a routine examination for the feasibility of the operation, including complete blood count (CBC), lever function test (LFT), Renal Function Test (RFT), and thorax X-ray. The entire results of the examination were normal. History of the patient’s general health was good. There were no comorbidities found in medical examination other than cleft palate. The results of laboratory tests and electrocardiogram were normal.

**Figure 1:**
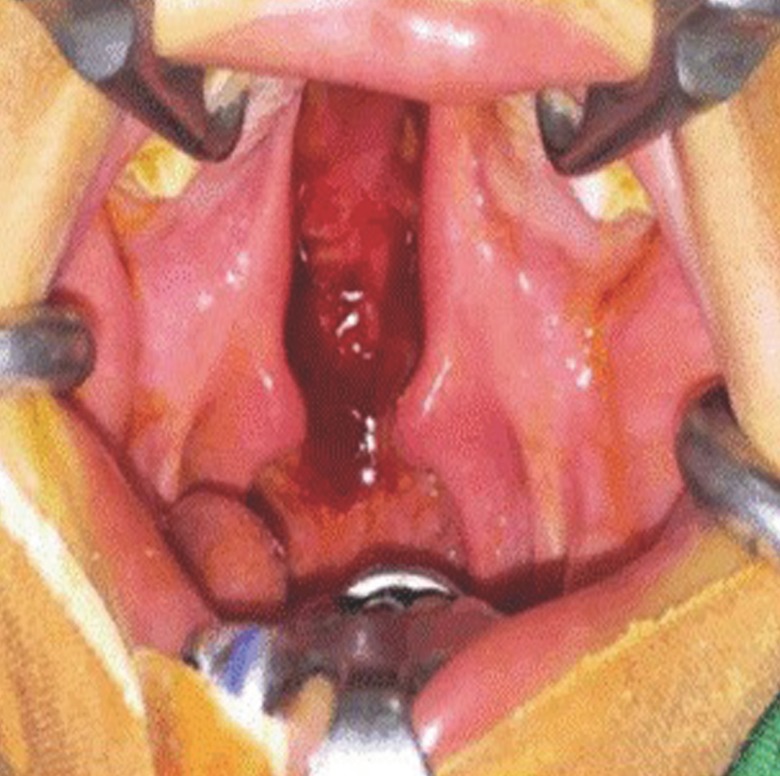
Clinical view of palatal cleft bilateral show 1-2 cm of defects.

**Figure 2:**
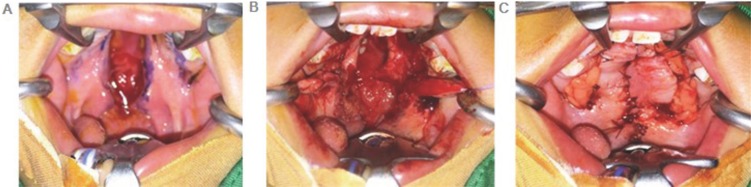
Operation procedure (a) Flap design. (b) The BFP was taken using blunt dissection and then placed toward the area of the defect and sutured with light pressure to the edge of flap and left open for the epithelialization process. (c) Suturing was performed with interrupted techniques.

All patients with 1–2 cm defects underwent surgery (Fig. 1). Palatal cleft closure was performed using the BFP as a pedicled graft for lateral relaxing wound closure on the left and right. Before the surgery, flap design mark was made and retrieval source BFP (Fig. 2a).

The BFP was taken using blunt dissection and then placed toward the area of the defect and sutured with light pressure to the edge of flap and left open for the epithelialization process (Fig. 2b). Suturing was performed with interrupted techniques using 4.0 vicryl thread (Fig. 2c).

Post-operatively, maintenance of oral hygiene every day on the first week was recommended to avoid infection. We performed oral hygiene by rinsing and cleaning the intra-oral cavity to prevent possible infections.

After surgery, post-operative control was performed in the first month to evaluate the outcome of surgery including wound healing, complication rate and post operative pain. All patients had a mean follow-up of 3.7 weeks with a minimum follow-up time of three weeks and a maximum follow-up of four weeks.

**Figure 3:**
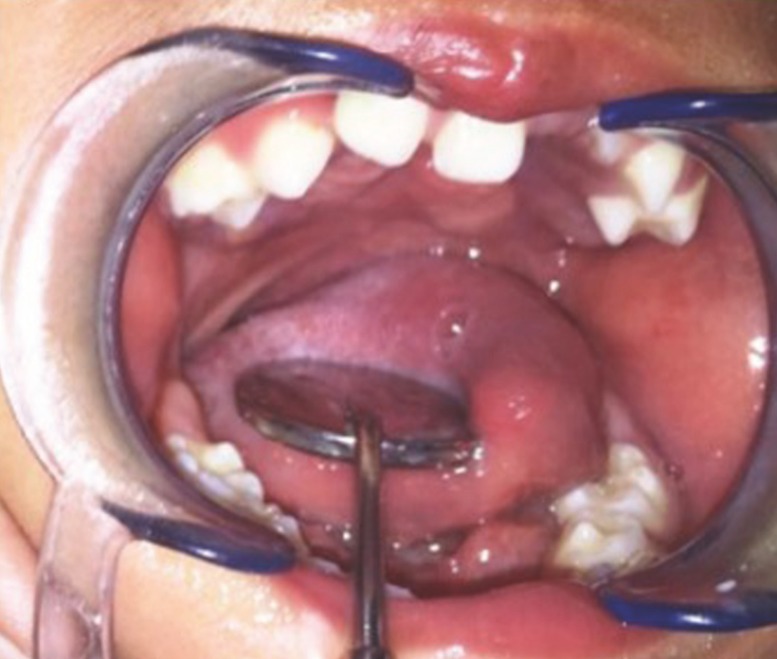
One month after surgery with the newly formed tissue and the surrounding tissue was recovered.

In the immediate postoperative period, most of the patients fared well. The palatal cleft was closed perfectly with the newly formed tissue and the surrounding tissue was recovered (Fig. 3). All patients who had an uneventful immediate postoperative period showed signs of BFP epithelialization characterized by a yellowish tissue beginning in the first week and ending within 3–4 weeks after surgery.

No patients showed specific intra or early postoperative complications correlating to the use of BFP or donor area, such as bleeding, infection, necrosis, palatal fistula, or paresthesia. There were no visible signs of dehiscence and none of the patients required additional surgery.

## Discussion

Our paper reports on cases of patients undergoing primary palatoplasty surgery that involved BFP filling in the lateral relaxing wound. We present various age of patients including infants to adults in order to perceive the use of BFP for closure the lateral wound on a larger defect size. These cases are a pilot study reported in Indonesian population.

Since Indonesia - as a developing country - has a considerable amount of low economic population with poor oral hygiene maintenance, this can lead to a serious and possibly fatal complication over the result of conventional palatoplasty procedure such as infection, wound dehiscence, risk of fistula, scarring, hematoma and partial necrosis (5,9,10). Due to this fact, one of the main consideration when planning surgical procedures using BFP as pedicled graft in our cases is the excellent healing of BFP with minimal complication.

On all patients in this report, raw surface closure was performed by using double opposite z-plasty (Furlow’s technique), and obtained satisfying result without discovering fistula formation. These results were supported by study from Levi *et al.* (4) who use of BFP as a graft with Furlow’s technique. The BFP is expected to provide a vascular tissue on open bone tissue, so that the risk of post operative complications can be reduced (1,10).

In this report, the failure of the procedure and the complication seen was very low. We only found mild pain and reasonable swelling in the cheek with spontaneous regression after 5 days. Clinically, the oral surface exposed yellowish–white fat revealed 3 days after surgery gradually became red within a week. At that time, the graft is covered with healthy looking oral mucosa. To consider this as a success, the epithelialization sign of the graft and the definite covering of the defect were the criteria that were taken into account.

In some cases in our patients, complete epithelization of the BFP was observed after 4 weeks of inset. Rapidis *et al.* (11) reported that the transferred BFP starts to epithelialize in a week and completes within 6 weeks. In separate articles, previous studies reported a complete epithelialization period of 4–6 weeks (1,3,5-8). The fast healing process in this BFP graft was associated to the progression of epithelialization (1,5,9). This process possibly come from rich blood supply and fresh wound around the defect (1,8,9,12).

Free graft of BFP could be a right option for closure of the defect in the maxillary region, however it may be insufficient in some cases (9,13). In our cases, the criteria for selecting the BFP was the availability of BFP regardless of the body weight and fat distribution. According to the literature, the average weight of each fat pad is approximately 9.3 gm and its average volume is 9.6 ml with a mean thickness of 6 mm (1,7,13). In infants and children, the BFP is abundant; however, the size is practically steady among other people (1,7).

In the series of our cases, we use the BFP as a pedicled graft for raw surface closure in palatoplasty procedure as it is a simple and fast procedure. The BFP retrieval required no special techniques and could provide plenty of tissue. These findings are consistent with previous published study, found that harvesting of the BFP requires less donor-site dissection and causes minimal donor-site morbidity (4,14,15). Galletti *et al.* (16) stated that the advantages of the BFP in the pedicled form are simple collecting and easy techniques, which thus appears as a good treatment choice, affording optimum results.

Another benefit of BFP utilization over the other options is the size of the defect, the free graft of BFP with low failure flap rate could be a good option for closure of defect in palatal region in small to medium -sized defects (5,9,12,13). In this series we encountered two cases in which the BFP was barely large enough to cover a defective area of 1.8 cm x 2 cm in size that reached a good healing. In another case, Grobe *et al.* (6) used BFP for reconstruction of defects which had the maximum size of 2 cm x 2 cm x 1 cm. Their study confirmed the predictable healing of BFP as well.

A clinical study conducted by Zhang *et al.* (3) observed the impact on maxillary growth in cleft palate patients based on lateral skull X-ray slices and upper dental models measurement, they found that patients who were undergoing reparative surgery with buccal fat pads filling in the bilateral raw bony surfaces had a decreased area of palate scar tissue and a reduced effect of the post-operative scar on maxillary growth. Our series is limited in this scope due to the short-term follow-up and rural residence of some patients. Another limitation of this pilot study is a small series with a large range of patients which might not apply to all patients equally. However, we clearly demonstrated the success of the application of BFP based on epithelialization sign observed in clinical assessment supported the existing literatures.

In conclusion, aside from the shortcomings of the current study, we conscientiously conclude that application of the bilateral BFP for closure the raw bony surfaces can provide satisfying results even when apply in wider defect in various age of patients. Further, our findings suggest that Indonesian cleft surgeons should consider in adopt this innovative technique for their cleft palate cases. None the less to sum up a firm conclusion on the size of the defect and its incidence of complication, a prospective study including a large series of patients from infants to adults is mandatory.
